# Primary breast osteosarcoma: A case report and review of the literature

**DOI:** 10.1002/ccr3.5044

**Published:** 2021-11-16

**Authors:** Ramesh Omranipour, Fereshteh Ensani, Maryam Hassanesfahani

**Affiliations:** ^1^ Department of General Surgery and Surgical oncology Tehran University of Medical Sciences Tehran Iran; ^2^ Breast Disease Research Center Tehran University of Medical Sciences Tehran Iran; ^3^ Department of Pathology Tehran University of Medical Sciences Tehran Iran

**Keywords:** breast malignancy, breast osteosarcoma, extraskeletal osteosarcoma, sarcoma

## Abstract

Primary breast osteosarcoma (PBOS) is an extremely rare and poor prognostic malignancy that has not a definitive treatment guideline. Here, we presented a successfully treated case of PBOS and provided a comprehensive review of the literature which revealed the divergence of opinions regarding the histogenesis and management of this malignancy

## Graphical abstract

Any suspicion for PBOS needs a comprehensive pathological evaluation including IHC and excluding metaplastic carcinoma as well as phyllodes tumor. Then, it should be managed similar to an extra‐skeletal sarcoma consisting of surgery or chemoradiation.

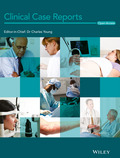

## 1 INTRODUCTION

Primary breast sarcomas are very rare, and primary breast osteosarcoma (PBOS) is still far less common. To the best of our knowledge, less than 150 cases of PBOS have been reported by this time; however, based on recent investigations, it is possible that even many of those cases were not really PBOS and they were some variants of metaplastic breast carcinoma. Indeed, they were considered as PBOS because of the lack of conducting a comprehensive histological and immunohistochemical (IHC) evaluation.^1^ Since there is no common consensus regarding the management of this specific kind of malignancy, reporting each case and its challenges could be helpful to provide more information about this type of aggressive and poor prognostic tumor.

## 2 CASE PRESENTATION

A 48‐year‐old otherwise healthy Caucasian woman presented with 2‐week history of a painless lump in the right breast. There was neither history of trauma nor chest wall irradiation, nor a previous history of a benign or malignant lesion in the breast. No screening mammography had been performed by that time. Physical examination revealed an ill‐defined, firm, mobile, and nontender 6 cm mass in the central and lateral portion of the breast, behind the nipple‐areola complex.

The physical exam of the axillary area and contralateral breast were unremarkable.

**FIGURE 1:**
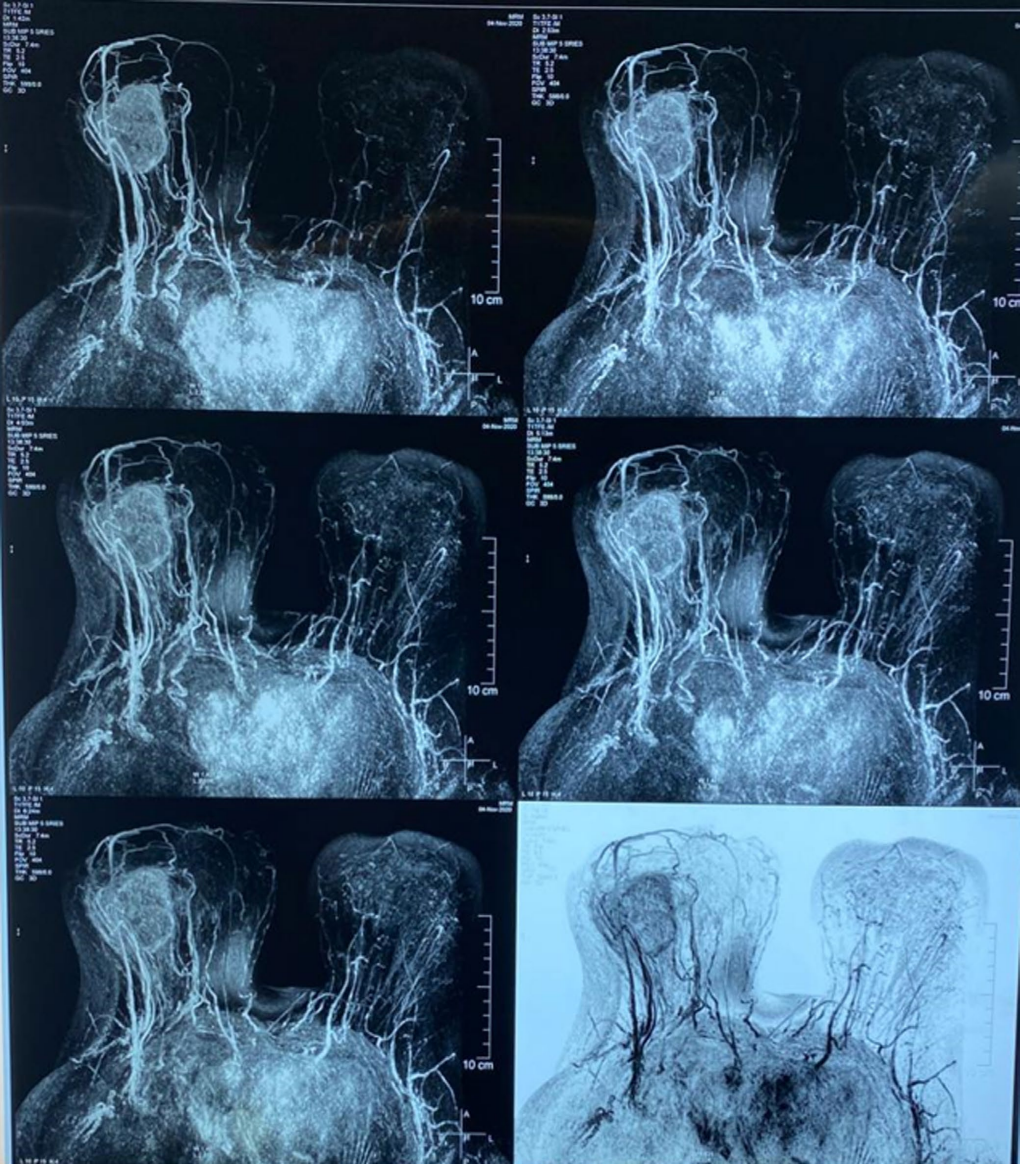
Breast MR

The mammography and ultrasonography revealed an irregular bulky mass with a lobulated border in the lateral part of the right breast. On MRI study, there was a 4‐cm mass in the lateral part of the right breast with a high signal intensity at the periphery of the tumor (Figure 1).

The initial core needle biopsy findings were compatible with malignant mesenchymal tumor with osseous differentiation which was confirmed by the second opinion. The tumor was composed of large atypical cells embedded in delicate eosinophilic vermiform plexus—which was questionable for osteoid and on IHC it was strongly positive for vimentin with 30%–35% proliferative activity (Ki 67) and nonreactive for Pan‐Ck. However, the definite diagnosis postponed to complete excision of tumor to exclude the possibility of metaplastic breast carcinoma or phyllodes tumor with osteosarcomatous component.

There was no evidence of distant metastasis based on liver function test, chest and abdominal CT, and bone scan.

As our institute routine, the patient was discussed in a multidisciplinary team and a simple mastectomy followed by Adriamycin and Ifosfamide regimen of chemotherapy and 50 Gy radiation was planned for her.

**FIGURE 2:**
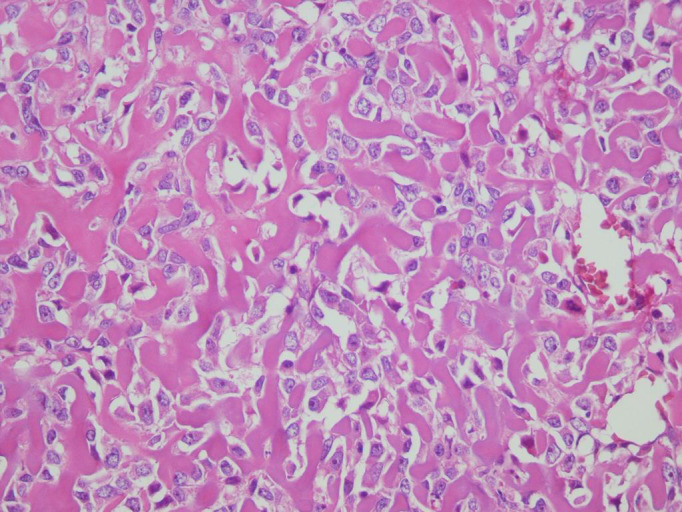
The highly atypical tumor cells embedded in a vermiform plexus of eosinophilic material (osteoid) 10 × 40 H&E

**FIGURE 3:**
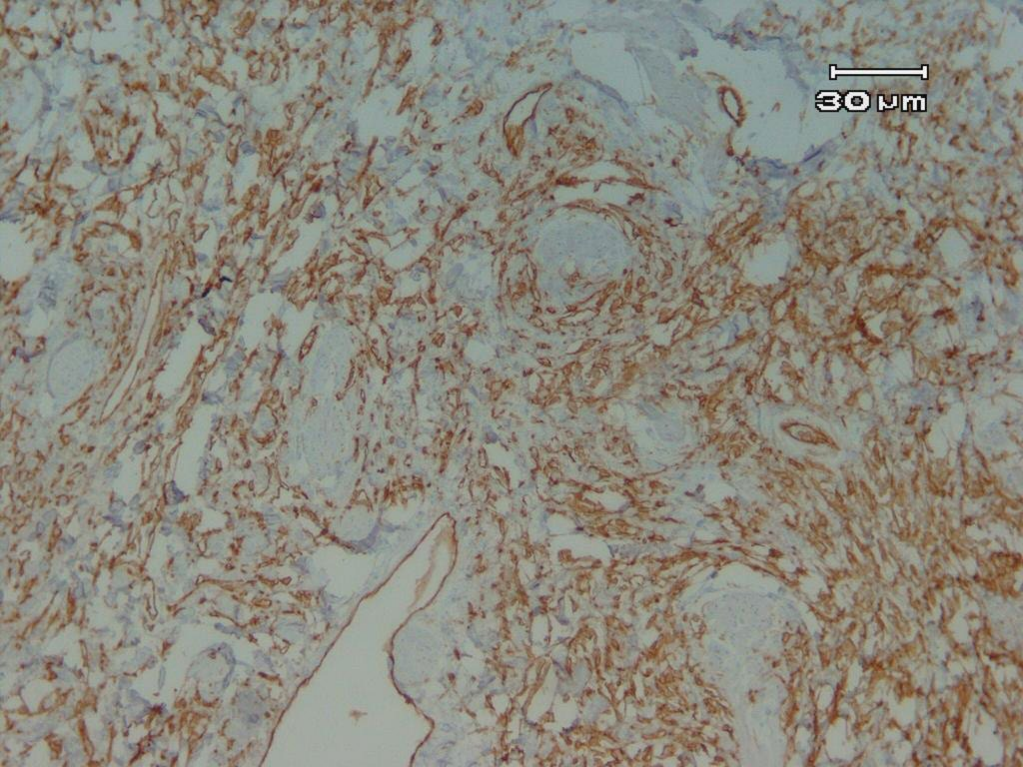
Positive immunostaining of tumor for vimentin (10 × 40)

The patient underwent a simple mastectomy. Although the tumor was not grossly fixed to the underlying chest wall structures, the deep margin was too close to the fascia of the pectoralis muscle. Therefore, a thin discoid shape layer of pectoral muscle just beneath the tumor lodge was resected en bloc with the rest of the specimen. Additionally, four enlarged lymph nodes were resected as a caution. All parts of the specimen were sectioned into 4‐μm thick and were stained with hematoxylin and eosin. Histological evaluation of the surgical specimen showed atypical tumor cells that were embedded in extensive ossified vermiform plexus of osteoid, bearing necrosis, and autolysis. There was no chondroid differentiation nor evidence of phyllodes tumor or metaplastic carcinoma (Figure 2). The histological result was confirmed by IHC with the avidin‐biotin‐peroxidase complex method leading to negativity for Pan‐CK and CAM 5.2 as well as strong positivity for Vimentin in addition to 30%–35% proliferative activity (Ki67; Figure 3). All margins and lymph nodes were free of tumor. It is worth mentioning that all the histological evaluations have been done by two expert pathologists in the field of sarcoma separately.

After recovery, the patient underwent chemotherapy with the mentioned regimen for five cycles and subsequently received radiotherapy 50 Gy. The treatment plan finished uneventfully last month.

## 3 DISCUSSION AND REVIEW OF THE LITERATURE

### 3.1 Epidemiology and incidence

Primary breast sarcomas accounting 0.0006%–1% of all breast malignancies and PBOS is far less common which accounts for about 4%–12.5% of primary breast sarcomas.^2^, ^3^

There is a discrepancy among the reports in the literature as three large studies of major referral centers over the same span of 40–50 years reported different incidences.^2^, ^3^, ^4^ Two studies from Md Anderson^4^ and Mayo clinic^3^ reported one to two cases of primary breast osteosarcoma among all sarcomas that they encountered in the same time window (almost 40–50 years); Meanwhile, the third study from Armed Forces Institute of Pathology in Washington DC has reported a much higher incidence of 50 cases within the almost the same period of 40 years.^2^

Although PBOS has been reported in a wide range of ages from 16‐year‐old teens^5^ to 96‐year‐old patients,^6^ it usually affects menopaused women in their sixties to eighties.^2^, ^3^, ^4^

### 3.2 Predisposing and precipitating factors

The previous history of burn has been noted in one case of PBOS^6^ but to the best of our knowledge, it has not been reported elsewhere. Prior history of epithelial breast cancer in the same side or contralateral side has been reported several times.^7^, ^8^ Some reported cases develop PBOS after irradiation.^9^, ^10^ In addition, some cases presented with a history of trauma or even a foreign body.^2^

### 3.3 Presentation

On almost all occasions the patients present with a palpable lump; in addition, they usually present as a slowly growing painless lump^2^, ^3^, ^4^even about 6 years,^11^ it has reported as a rapidly enlarging mass.^12^ Regarding the size, it can be as large as 30 cm^2^ or 12 cm^3^ but the average of their size is around 5.5^3^

Sites of origin: before labeling them as a primary mammary gland tumor, other neighbor's origins should be ruled out such as underlying ribs, sternum, and even the pectoralis muscle which has been reported by Orta et al.^13^ It is worth mentionin that there is always a possibility for breast osteosarcoma to be a metastasis from primary bone osteosarcoma.^14^, ^15^

### 3.4 Histogenesis

We believe that not only the rarity of PBOS but also not having enough evidence and consensus about its histogenesis have led to the lack of agreement among the management. Therefore, here we will review some of the literature in detail in order to talk about the histogenesis and the origin of the PBOS. In 2000, Hellmen et al^16^ conducted an animal study on dogs and showed that mammary spindle cell tumors and osteosarcomas are derived from pluripotent stem cells. In this regard, some authors believe PBOS is originated from totipotential stem cells in the mammary gland^2^, ^16^ as it can happen after radiation to the chest wall without any significant previous lesion of trauma.^9^, ^10^ In a different circumstance, there are other studies that mentioned the origin of these specific types of breast sarcoma can be the result of metaplasia, either metaplastic transformation in a pre‐existing malignant lesion or non‐malignant ones.^17^, ^18^, ^19^, ^20^ Meanwhile, there are some papers that emphasized the type of pre‐existing lesion such as an epithelial cancerous lesion,^1^, ^21^ an intraductal papilloma,^22^ previous existing of a fibroadenoma,^2^, ^23^ or more frequently, phyllodes tumor has been introduced as the pre‐existing lesion.^24^, ^25^, ^26^, ^27^, ^28^, ^29^, ^30^ Diversely, there is a comprehensive investigation that has been published in 2012 by Emad A. Rakha^1^ that showed almost all PBOS are derived from the epithelial origin after being under the metaplastic transformation.

### 3.5 Diagnostic workup

There is no doubt regarding the importance of mammography and sonography as the first steps of evaluation of a breast lump. However, as usual, these tumors would present similar to the benign lesions on modalities,^2^, ^31^ some other evaluation may be needed. In the case of evidence for PBOS on core needle biopsy, in addition to the routine workup of breast cancer, some other assessments such as bone scan and serum alkaline phosphatase activity are recommended by some authors.^32^, ^33^, ^34^ Furthermore, there is a suggestion to use serum alkaline phosphatase activity for follow‐up and monitoring purposes.^34^ MRI has been used for additional evaluation and information; meanwhile, Dynamic Contrast‐Enhanced Magnetic Resonance Imaging, Diffusion‐Weighted Imaging Findings, and Proton Spectroscopy have revealed novel findings.^35^

### 3.6 Treatment

There is no general and comprehensive consensus on the management of PBOS. Some consider it as a sarcoma and emphasized that the management of PBOS should be similar to that of other sarcomas^20^, ^36^; whereas, some others believe that it should be treated like triple‐negative epithelial carcinomas.^1^ The value and effectiveness of chemotherapy have been emphasized, particularly with the tumor size more than 5 cm^37^, ^38^, ^39^; however, there are still some reports that they did not offer chemotherapy to/for their patients^22^, ^31^ even with the tumor size of 6 cm.^39^ Although Axillary Lymph Node Dissection (ALND) or sentinel lymph node biopsy has been performed in some reports^18^, ^22^, ^23^, ^40^, ^42^, ^43^ in most of the reports ALND was not performed for the patients.^2^, ^3^, ^4^, ^24^, ^41^, ^42^, ^43^ Similar to chemotherapy, considering irradiation as a part of treatment is not widely accepted at least as far as the tumor size is not large enough and the margins are clear from tumoral deposits^4^, ^31^, ^37^; however, chest wall irradiation has been suggested by some other authors to reduce the risk of local recurrence as a routine part of treatment.^44^ It seems that achieving a negative margin either with wide local excision or simple mastectomy without ALND is widely accepted/adapted and offering chemotherapy and radiotherapy should be based on prognostic factors of each patient. It is worth mentioning that due to the local recurrence and distant metastasis rate of about 40% within the first year,^2^, ^45^ an aggressive approach including appropriate surgery and adjuvant therapy should be considered while administration of chemotherapy or radiotherapy must be balanced against the consequences of these treatments per case.

### 3.7 Prognostic and predictive factors

The estimation of 5 and 10‐ year survival is 38% and 32% respectively.^2^ For overall survival, among many factors such as patient's age at the presentation, tumor size, histopathologic grade and subtype, atypia, mitotic activity, type and extent of surgery, and surgical margin status, only the tumor size less than 5 cm and fibroblastic subtype have been more accepted as favorable factors and most reliable ones among many of the series.^1^, ^2^, ^3^, ^46^ Furthermore, surgical margin status and the type of surgery (local excision vs mastectomy) can be predictive factors for local recurrence. The most common sites for metastasis are the lung, bone, skin, and heart consequently.^2^, ^20^, ^26^, ^39^, ^47^

## 4 CONCLUSION

Primary breast osteosarcoma is an extremely rare malignancy with divergence among authors' opinions regarding its histogenesis and management. Uncertainty about the origin of PBOS and whether it is a metaplastic transformation of epithelial cells or pre‐existing lesions such as phyllodes tumors or even arising directly from totipotential stem cells are part of the scenario that has made the dilemma even worse.

In addition, there is not a comprehensive and widely accepted management guideline for its management. Some of the cases received all three parts of possible treatments including surgery, chemotherapy, and radiotherapy; however, there are so many cases that received only one item.

We believe that the most important reason for this discrepancy is not having an agreement about the origin of the tumor; as a result, many authors have approached this tumor similar to a sarcoma while others have tried to manage it like an epithelial‐originated malignancy.

Using a comprehensive IHC panel and meticulous pathological evaluation of the tumor would be helpful to find the best plan of treatment meanwhile it is worth mentioning that reporting each case and publishing them would be valuable to collect more information about this specific type of malignancy.

## CONFLICT OF INTEREST

The authors declare that there is no conflict of interest.

## AUTHOR CONTRIBUTIONS

All the authors contributed to manuscript preparation and critical revision.

## ETHICAL APPROVAL

Applicable.

## CONSENT

Written informed consent was obtained from the patient.

## Data Availability

The data that support the findings of this study are available on request from the corresponding author. The data are not publicly available due to privacy or ethical restrictions.
